# Impact of 719Trp>Arg Polymorphism of KIF 6 Gene on Contrast Induced Nephropathy in Patients Undergoing Coronary Angiography or Percutaneous Coronary Intervention

**DOI:** 10.5334/gh.1105

**Published:** 2022-02-28

**Authors:** Lucia Barbieri, Monica Verdoia, Harry Suryapranata, Stefano Carugo, Giuseppe De Luca

**Affiliations:** 1 Fondazione IRCCS Ca’ Granda Ospedale Maggiore Policlinico, Division of Cardiology, Department of Clinical Sciences and Community Health, University of Milan, IT; 2 Division of Cardiology, Nuovo Ospedale degli Infermi, Biella, IT; 3 Division of Cardiology, Azienda Ospedaliera-Universitaria ‘Maggiore della Carità,’ EasternPiedmontUniversity, Novara, IT; 4 Radboud University Medical Center, Nijmegen, NL

**Keywords:** KIF 6 polymorphism, contrast induced nephropathy, interventional cardiology

## Abstract

**Background:**

The identification of preventive strategies, such as statin therapy, is crucial for the management of contrast-induced nephropathy (CIN). Several studies showed the association between KIF6 polymorphism (replacement of Trp719 with Arg) and an increased cardiovascular risk, while others showed a correlation between ‘pleiotropic’ effects of statins and a reduction in cardiovascular events in the population with the risk allele due to the documented modulation of response to statin by KIF6 polymorphism. Aim of this study is to assess the impact of KIF6 polymorphism on the development of CIN.

**Methods:**

We analysed 1253 consecutive patients undergoing coronary angiography/PCI. Serum creatinine was collected at baseline, 24 and 48 hours after contrast exposure. We identified the different allelic patterns and assessed the incidence of CIN (absolute increase of 0.5mg/dL or relative >25% in creatinine at 24 and 48h).

**Results:**

KIF6 Arg mutation was found in 669 patients (heterozygotes n = 525, homozygotes n = 144). The total prevalence of CIN was 12.5% and we did not find any association between KIF6 polymorphism and CIN development (11.3%, 13.7%, 13.2% p = 0.30). At subgroups analysis among statin ‘naïve’ patients we found a higher prevalence of CIN in homozygous patients as compared to wild-type (20.7% vs 11.3%, p = 0.05), while opposite results were observed among patients with statin therapy (8.6% vs 13.2%, p = 0.28).

**Conclusion:**

KIF6 homozygous Arg was associated with a significant increase in the risk of CIN only among statin naive patients. Future studies are needed to evaluate the beneficial effects of statin especially in this subset of patients.

## Introduction

Coronary artery disease (CAD) is a multifactorial and complex condition resulting from the interaction between genes and environmental factors. Statin treatment plays a central role in the prevention of cardiovascular disease, in fact, in addition to their impact on cholesterol levels, they have shown multiple non lipid-lowering pleiotropic effects such as anti-oxidant, anti-inflammatory and anti-thrombotic properties with enhancement of endothelial nitric oxide production and reduction of endothelin secretion [1,2,3]. Genetics plays an important role in determining the inherent CAD vulnerability and in affecting the response and potential benefic effects of statin therapy. Contrast-induced nephropathy (CIN) is a common complication of procedures that foresee the use of contrast media and is known as the third leading cause of hospital-acquired acute kidney injury, accounting for 11% of all cases [4]. In the last decades several therapies for the prevention of CIN, such as different hydration and alkalinization measures [5,6], N-acetylcysteine (NAC) [7], Fenoldopam, dialysis and hemofiltration [8,9], have been explored in randomized clinical trial with conflicting results. Due to the important pleiotropic effects of statins, a large number of studies have assessed their role for the prevention of CIN. Recent studies showed a protective effect of statin therapy in patients treated before coronary angiography/PCI [10,11], while other studies showed that statin therapy did not provide beneficial effects in the prevention of CIN [12,13].

Kinesis-Like Protein 6 (KIF 6) is an omodimeric protein expressed in coronary arteries and other vascular tissues, that is involved in cellular microtubular transport [14]. The impact of KIF 6 gene on cardiovascular risk modulation has been investigated since 2007 due to the presence of a single nucleotide polymorphism (non-synonym replacement A>G) related with the replacement of Trp719 with arginine (Arg). Several prospective trials and meta-analysis [15,16,17] assessed the association between this genetic variant (expressed in 60% of the European population) and a significant increase in cardiovascular risk. These results were not confirmed in Heart Protection study, a large analysis that involved more than 18000 patients [18]. Several mechanisms such as a modification in the particles binding capacity [19], a modulation in the endothelial cells progenitors growth [20], and an increased expression of KIF 6 in the population with the risk allele [21] has been proposed to explain this association. Particular attention in last years has been focused on the role of Trp719Arg polymorphism in the modulation of response to statin treatment. Several clinical trials showed a significant association between anti-inflammatory, metabolic and vasoprotective effects of statin therapy and a reduction in cardiovascular events in the population with the risk allele [22,23]. Moreover, these protective effects with a significant reduction (about 13%) of LDL-cholesterol level and of the risk of cardiovascular events has been recently confirmed by two meta-analysis [24,25]. Therefore, the aim of the present study was to assess the association between KIF-6 polymorphism (Trp719Arg polymorphism) and the occurrence of CIN and modulation of the protective effects of statin therapy.

## Material and Methods

Clinical, demographic and procedural data of consecutive patients undergoing coronary angiography and/or PCI were collected in our dedicated database protected by password. Patients with impaired renal function at baseline (creatinine clearance <60ml/min) were treated with standard hydration (1ml/kg/h of saline solution 0.9% 12h before and after the procedure or with saline solution 0.5ml/kg/h, if ejection fraction ≤40% or with sodium bicarbonate received 3 ml/kg for 1h before contrast exposure followed by an infusion of 1 ml/kg/h for 6h after the procedure). CIN was defined as an absolute increase of 0.5mg/dL or a relative increase >25% in serum creatinine levels at 24 and 48h after the procedure.

### Genetic analysis

A blood sample for the determination of Trp719Arg polymorphism was collected for all patients. We therefore performed DNA extraction by the use of Sigma Aldrich Gen Elute system for each patient. We set the polymerase chain reaction (PCR) with reagents concentration optimization and MgCl2 concentration curve. Amplification of the region of interest with PCR and consequent electrophorethic run on agarose gel and digestion with restriction enzyme Fok I was performed for each sample. Digestion product underwent another electrophorethic run on agarose gel and subsequent analysis with UV scan. We therefore were able to identify the different allelic patterns of our population.

### Biochemical measurements

Blood samples were drawn at admission in patients undergoing elective (following a fasting period of 12 h) or urgent coronary angiography. Glucose, creatinine, uric acid, blood cells count and lipid profile were determined by standard methods. Creatinine was measured at 12, 24 and 48 hours after the procedure or longer in case of development of CIN.

### Coronary angiography

Coronary angiography was routinely performed by the Judkins technique using 6-French right and left heart catheters. Angiographic approach was at the discretion of the interventional cardiologist, but in last five years our standard approach is the radial one in more than 95% of cases. Quantitative coronary angiography (Siemens AcomQuantcor QCA, Erlangen, Germany) was performed by two experienced cardiologists who had no knowledge of the patients’ clinical information. Significant coronary artery disease was defined as at least one coronary stenosis more than 50%. The contrast medium used was non-ionic, low osmolality (Optiray-Ioversol, 350mg/ml, Ultravist-Iopromide, 370mg/ml, Visipaque-Iodixanol, 320mg l/ml).

## Statistical analysis

Statistical analysis was performed with the SPSS 17.0 statistical package. Continuous datas were expressed as mean ± SD and categorical data as percentage. Analysis of variance and the chi-square test were used for continuous and categorical variables, respectively. Patients were subsequently divided in three groups according to the genetic profile. The impact of KIF 6 polymorphism on the occurrence of CIN and its relationship with statin therapy at admission was assessed among three groups (wild type, heterozygotes, homozygotes) and two groups (wild type, polymorphic) according to the genetic status. Multiple logistic regression analysis was performed to evaluate the relationship between KIF 6 polymorphism and the development of CIN, after correction for baseline confounding factors (clinical and demographic variables with a p value <0.05), that were entered in the model in block.

## Results

**Figure 1:**
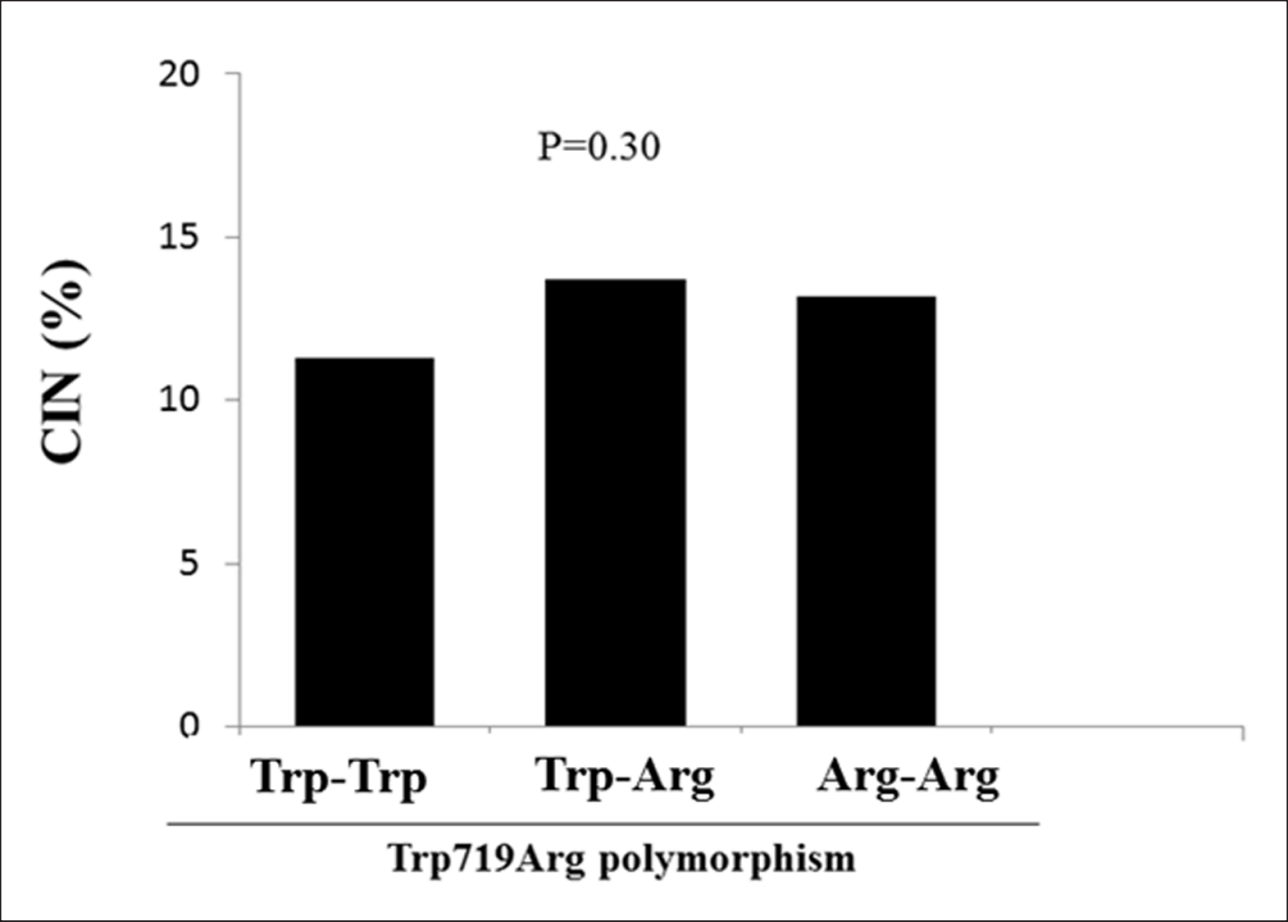
Bar graph showing the relationship between KIF 6 polymorphism and the risk of contrast-induced nephropathy.

**Figure 2:**
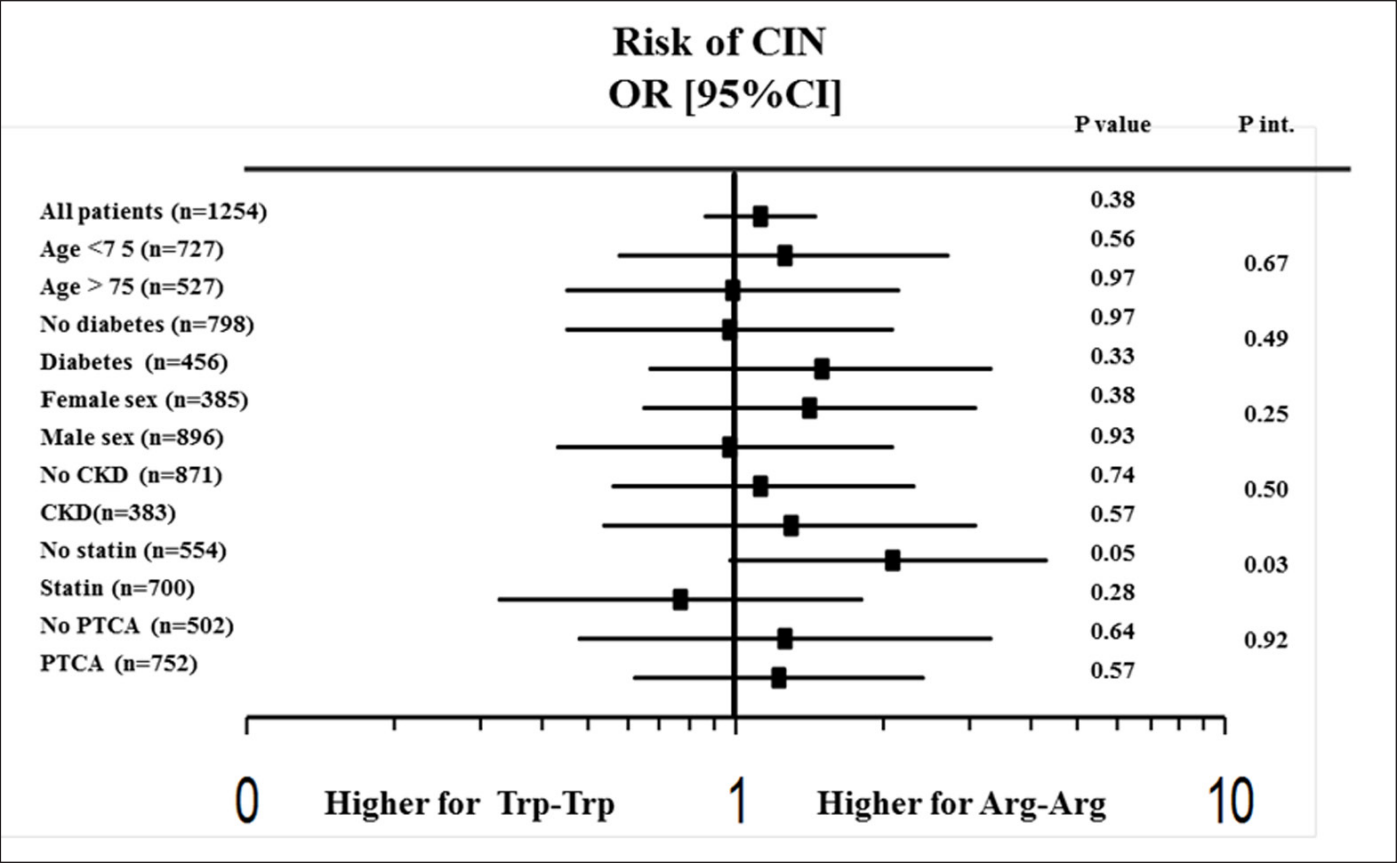
Forrest plot showing the relationship between KIF 6 polymorphism (wild type and homozygous patients) and CIN among main risk known risk factors.

**Figure 3:**
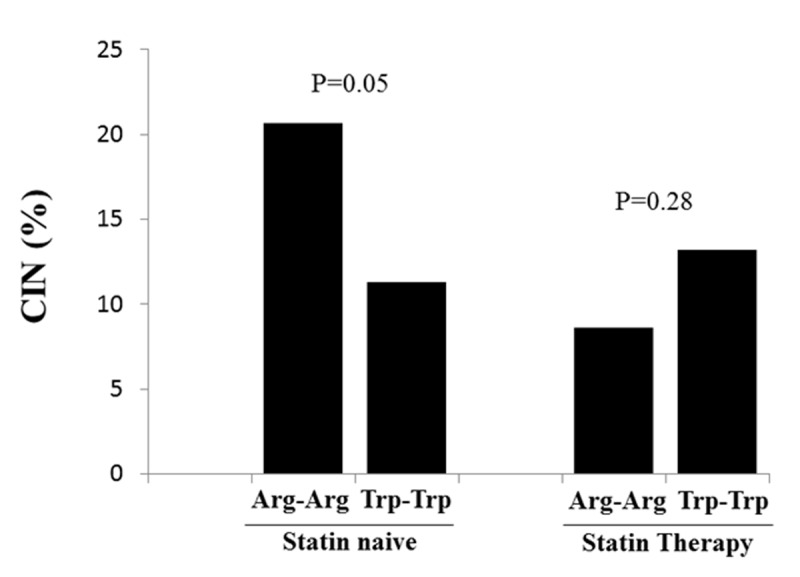
Bar graph showing the relationship between statin therapy at admission and the risk of contrast-induced nephropathy among homozygous and wild-type patients.

We analysed a total of 1253 patients undergoing coronary angiography and/or angioplasty. KIF 6 Arg mutation was found in 669 patients (heterozygotes n = 525, homozygotes n = 144). Our population respected Hardy-Weinberg equilibrium (p = 0.14). Patients were divided into three groups according to the genetic status (wild type n = 584, heterozygotes n = 525, homozygotes n = 144). Baseline clinical and demographic characteristics, indication for angiography, procedural characteristics, baseline chemistry and admission therapy are listed in ***Table 1***. Wild type patients were more often in therapy with angiotensin receptor blockers (ARB) (p = 0.01), beta blockers (p = 0.04), but less with acetylsalicylic acid (ASA) (p = 0.03) and clopidogrel (p = 0.01) at admission and they had higher haemoglobin levels (p = 0.03). No other significant differences were found. The total prevalence of CIN in our population was 12.5% and we did not find any significant association between KIF 6 polymorphism and its development (Group 1 11.3%, Group 2 13.7%, Group 3 13.2% p = 0.30) (***Figure 1***). This result was confirmed by multivariate analysis after correction for baseline confounding factors (Adjusted OR [95%CI] = 1.12 [0.86–1.46], p = 0.38). Similar results were found dividing our population in two groups according to the presence of the polymorphism (wild type 11%, Polymorphic 13.7%, p = 0.19). At subgroups analysis no impact of homozygous KIF 6 polymorphism was observed on CIN development according to major risk factors such as diabetes (p int 0.49), renal failure (p int 0.50), gender (p int 0.25), older age (p int 0.67) and PCI (p int 0.92) (***Figure 2***). Interestingly among statin ‘naïve’ patients we found a higher prevalence of CIN in homozygous patients as compared to wild-type patients (20.7% vs 11.3%, p = 0.05), while opposite results were observed among patients with statin therapy at admission (8.6% vs 13.2%, p = 0.28) (p int 0.03) (***Figure 3***).

**Table 1:** Clinical and procedural characteristics of patients according to KIF 6 polymorphism.


VARIABLE	KIF 6 POLYMORPHISM			

**Clinicalcharacteristics**	Trp-Trp (n = 584)	Trp-Arg (n = 525)	Arg-Arg (n = 144)	p-value

Age (M-SD)	68.1+/–10.7	67.8+/–11	68+/–12.3	0.90

Male sex (%)	68.2	71.6	65.3	0.94

Hypertension (%)	75.9	72.2	80.6	0.81

Smokers (%)				0.26

Active smokers (%)	19.9	20.6	22.9	

Previoussmokers (%)	22.1	28.4	20.1	

Hypercolesterolemia (%)	58	56.2	66	0.31

Diabetes (%)	35.3	35.4	42.4	0.23

Family history of CAD (%)	26.6	27.4	33.3	0.17

Previous AMI (%)	27.2	24.8	27.1	0.66

Previous PCI (%)	26	28.2	28.5	0.40

Previous CABG (%)	10.9	12.8	12.5	0.40

Previous CVA (%)	6.8	6.9	6.3	0.85

Renal failure (%)	12.8	15.8	15.3	0.21

**Indication for angiography**				0.15

Stable angina or silent ischemia (%)	22.2	22.3	20.2	

Acute Coronary Syndrome (%)	55.9	59.9	67.7	

DCM or valvular disease (%)	22	17.8	12.1	

**Baseline Chemistry**				

Haemoglobin (M-SD)	13.5+/–1.6	13.3+/–1.7	12.9+/–1.6	0.03

Platelet (M-SD)	214.4+/–60.3	219.4+/–70.5	225.1+/–70.5	0.16

Glycaemia at admission (M-SD)	127+/–48.4	124.6+/–47.2	125.3+/–64.7	0.07

Baseline creatinine (M-SD)	1+/–0.28	1.05+/–0.45	1.01+/–0.36	0.08

Absolute creatinine increase (M-SD)	0.08+/–0.27	0.07+/–0.24	0.11+/–0.31	0.30

Relative creatinine increase (M-SD)	0.09+/–0.26	0.09+/–0.19	0.13+/–0.31	0.20

Creatinine clearance (M-SD)	78.8+/–31.6	78+/–34.6	79.7+/–34.2	0.83

Reactive protein C (M-SD)	1.34+/–3.02	1.33+/-2.43	1.36+/-2.46	0.99

Total Cholesterol (M-SD)	160.6+/–40.8	161.5+/–43.1	158.1+/–44.7	0.70

HDL-Cholesterol (M-SD)	41.6+/–13.4	41.2+/–13	41.5+/–13.9	0.84

Triglycerides (M-SD)	134+/–71.2	139.4+/–98.8	132.6+/–67.9	0.48

LDL-Cholesterol (M-SD)	93.3+/–36.3	93.6+/–39.9	90.8+/–38.7	0.74

**Procedural characteristics**				

PTCA (%)	58.6	61.3	55.2	0.89

Radial access (%)	33.2	36.2	36.1	0.32

Contrast volume (M-SD)	233.7+/–157	231.3+/–152.5	237.4+/–172.9	0.91

**Theraphy at admission**				

ACE I (%)	41.4	42.3	43.4	0.76

ARB (%)	26.9	20.2	19.2	0.01

Statins (%)	54.3	50.9	58.3	0.90

Nitrate (%)	35.8	39.2	36	0.58

Beta-Blockers (%)	57.7	57	46	0.04

ASA (%)	59.6	61.7	71.1	0.03

Clopidogrel (%)	20.8	26.4	29.3	0.01

CalciumAntagonist (%)	22.2	21.9	27.5	0.35

Diuretics (%)	35.2	32.1	36.8	0.82


SD = Standard Deviation; CAD = coronary artery disease; MI = myocardial infarction; PCI = percutaneous coronary intervention; CABG = coronary artery by-pass graft; DCM = dilated cardiomyopathy; HDL = high density lipoprotein; LDL = low density lipoprotein; PTCA = percfutaneous coronary intervention; ACE = angiotensin converting enzyme; ARB = angiotensin II receptor blockers; ASA = acetylsalicylic acid.

## Discussion

The main finding of our study is the presence of a significant statin therapy modulation among KIF 6 homozygous Arg patients and the development of CIN after coronary interventions.

The extensive management of coronary artery disease by PCI and the high-risk profile of patients currently treated [26,27] has driven a lot of interest on the occurrence of CIN, that is a common complication of procedures that foresee the use of contrast media and is known as the third leading cause of hospital-acquired acute kidney injury [28]. Great efforts have been done in last decades to identify new risk factors that foresee this complication and potential preventive strategies, such as pre-procedural high dose statin administration [29,30,31,32]. The role of KIF 6 gene on cardiovascular risk modulation has been investigated in last ten years due to the presence of a single nucleotide polymorphism (non-synonym replacement A>G) related with the replacement of Trp 719 with arginine (Arg). Several prospective trials and meta-analysis assessed the association between this genetic variant and the risk of cardiovascular disease, with conflicting results [15,16,17,18]. Particular attention has been focused on the role of the Trp719Arg polymorphism in the modulation of response to statin treatment. Several clinical trials showed a significant association between anti-inflammatory, metabolics and vasoprotective effects of statin therapy and a reduction in cardiovascular events in the population with the risk allele [22,23]. Moreover, these protective effects with a significant reduction (about 13%) of LDL-cholesterol level and of the risk of cardiovascular events has been recently confirmed by two meta-analysis [24,25]. Concerning our knowledge, no studies has so far addressed the impact of KIF 6 polymorphism on the development of CIN. Statins, in addition to their impact on cholesterol levels have been shown to possess multiple non lipid-lowering pleiotropic effects such as anti-oxidant, anti-inflammatory and anti-thrombotic properties with enhancement of endothelial nitric oxide production and reducing of endothelin secretion [2,3]. As well known from literature, the pathogenesis of CIN is the result of endothelial dysfunction, cellular toxicity from the contrast agent and tubular apoptosis resulting from hypoxic damage or reactive oxygen species [33], therefore, pharmacological prophylactic strategies based on antioxidant properties have been considered for its prevention. In our population we found that statin therapy at admission did not influence the development of CIN in overall population, while, there was a significant interaction between KIF 6 polymorphism, statin therapy and CIN development, suggesting that the nefro-protective effects of these drugs could be even more relevant in carriers of the KIF-6 variant allele. In fact, Arg homozygotes not treated with statins at the moment of angiography showed a significantly enhanced risk of CIN, that was reduced among patients treated with statin therapy at admission. Future large studies are certainly needed to confirm our finding, and further evaluate the modulation of the beneficial effects of statin therapies by KIF-6 gene polymorphism in the prevention of CIN.

## Limitations

A main limitation of our study was that we evaluated the occurrence of CIN at 48-72 hours, but this complication may appear even later than this time threshold. Furthermore, we were not able to provide data on clinical follow-up and in particular on the progression of kidney failure, being this disease chronically progressive.

## Conclusions

Our study showed among patients undergoing coronary angiography and/or PCI that KIF 6 homozygous Arg was associated with a significant increase in the risk of CIN only among statin naive patients. Future additional studies are certainly needed to confirm our findings and to evaluate the beneficial effects of statin therapy especially in this subset of patients.
